# Effects of the Hydration State on the Mid-Infrared Spectra of Urea and Creatinine in Relation to Urine Analyses

**DOI:** 10.1177/0003702816641263

**Published:** 2016-06

**Authors:** Katherine V. Oliver, Amandine Maréchal, Peter R. Rich

**Affiliations:** Glynn Laboratory of Bioenergetics, Institute of Structural and Molecular Biology, University College London, United Kingdom

**Keywords:** Attenuated total reflection Fourier transform infrared spectroscopy, ATR FT-IR, Mid-infrared spectroscopy, Mid-IR, Urea, Hydration, Creatinine, Medical diagnostics

## Abstract

When analyzing solutes by Fourier transform infrared (FT-IR) spectroscopy in attenuated total reflection (ATR) mode, drying of samples onto the ATR crystal surface can greatly increase solute band intensities and, therefore, aid detection of minor components. However, analysis of such spectra is complicated by the existence of alternative partial hydration states of some substances that can significantly alter their infrared signatures. This is illustrated here with urea, which is a dominant component of urine. The effects of hydration state on its infrared spectrum were investigated both by incubation in atmospheres of fixed relative humidities and by recording serial spectra during the drying process. Significant changes of absorption band positions and shapes were observed. Decomposition of the CN antisymmetric stretching (ν_as_) band in all states was possible with four components whose relative intensities varied with hydration state. These correspond to the solution (1468 cm^–1^) and dry (1464 cm^–1^) states and two intermediate (1454 cm^–1^ and 1443 cm^–1^) forms that arise from specific urea–water and/or urea–urea interactions. Such intermediate forms of other compounds can also be formed, as demonstrated here with creatinine. Recognition of these states and their accommodation in analyses of materials such as dried urine allows more precise decomposition of spectra so that weaker bands of diagnostic interest can be more accurately defined.

## Introduction

There is a rapidly increasing literature on applications of vibrational infrared spectroscopy to analyses of complex biological tissues and fluids in order to identify unique signatures of cell types, diseased tissues and diagnostic markers of specific diseases.^1–10^ The mid-infrared (MIR) spectra of such materials are inevitably complex combinations of many overlapping infrared (IR)-active components. Diagnostic features of interest are in general minor components of these spectra whose small IR intensities are usually overlapped by much stronger bands from more dominant components.

Biological samples, such as urine and thin tissue biopsy sections, are often dried before analysis.^4,7^ Drying removes the strong, broad absorption bands of water and, in the case of measurements in attenuated total reflection (ATR) mode, increases the IR intensities of all other components by maximizing their concentrations across the IR-active pathlength into which the evanescent wave penetrates. When applying analytical protocols to IR data obtained from dried versus hydrated materials, it is important to take into account the fact that the IR spectra of many materials are different in aqueous solution and the fully dried states. However, a further complication is that some materials can also exhibit additional distinct states during drying whose IR characteristics differ from both the solution and fully dried forms. This can be an important additional consideration when analyzing materials in which residual water levels are not easy to remove in a consistent manner since such intermediate states can become trapped within the material as it dries.

Urea, with its strong interactions with water, is a particularly clear example of such behavior. Its strong hydrogen bonding propensity results in its well-known chaotropic effects in aqueous solutions.^11^ Molecular modeling of its interaction with an increasing number of water molecules suggests that the first four water molecules form the primary hydration sphere and three more form the secondary hydration sphere.^12–14^ Additional types of urea–water complexes are also predicted.^12^ Such urea-bound water molecules have been detected directly in aqueous solutions of urea by polarization-resolved mid-IR pump-probe spectroscopy;^15^ bulk water molecules beyond the hydration sphere around urea are not significantly perturbed,^13,15–17^ although this has been questioned with nuclear magnetic resonance studies of long range order.^16^ At high concentration, urea may also form dimers or higher order structures,^12,13,18,19^ although several studies have questioned their existence.^17,20,21^ Some of these controversies may arise due to the different nature of the probe between different spectroscopic techniques. For an extensive review see Idrissi.^21^

Here, IR spectroscopy is used to demonstrate the different forms of urea and creatinine that can form at different hydration states. Urea and creatinine are two components of urine that are often measured clinically (using non-IR spectroscopic methods) and can be used as markers for urine concentration against which other components can be normalized. Several methods for quantitation of urea and creatinine in wet urine samples have been described that are based on their well-defined solution IR spectra.^6,9^ For example, as described here, urea concentrations in aqueous solution or in wet urine can be quantitated by fitting a Gaussian component to its strong ν_as_ (antisymmetric C–N stretch) band at 1468 cm^–1^.^22^ When urea is completely dry this ν_as_(CN) band shifts to 1464 cm^–1^. However, at least two additional states of urea with a ν_as_(CN) band at 1454 or 1443 cm^–1^ can be observed in partially hydrated urea. When whole urine is dried, the solid materials tend to trap these intermediate forms. Hence, accurate protocols for component decomposition, or for comparison of “wet” and “dried” biological fluids and tissues, should accommodate such additional states.

## Experimental

### Chemicals

Ammonium chloride (purity ≥ 99.8 %), potassium hydroxide (purity ≥ 85.4 %), potassium phosphate (purity ≥ 99.9 %) and sodium chloride (purity ≥ 99.5 %) were purchased from VWR International BDH, Leicestershire, UK. Creatinine (purity ≥ 98 %), potassium sulfate (purity ≥ 99 %) and urea (purity ≥ 99.5 %) were purchased from Sigma-Aldrich, Dorset, UK.

### Clinical samples

Five urine samples were collected from volunteer healthy donors at University College London and Imperial College London after obtaining informed consent. Urine samples were analyzed fresh. Urine sample composition varies greatly between individuals because it is heavily influenced by factors such as recent diet, hydration, body mass/composition and time of day. The average urea concentration of these samples was 202 mM (standard deviation: 102 mM), the average creatinine concentration was 5 mM (standard deviation: 3.7 mM) and no protein was detectable. These levels are within the expected ranges of healthy subjects.

### Data collection

Spectra were recorded with a Bruker IFS/66 S FT-IR spectrometer, fitted with a liquid nitrogen-cooled MCT detector and a KBr beamsplitter. Experiments were run in ATR mode using a 3 mm diameter silicon microprism with ZnSe optics (three reflections; SensIR). Data were recorded at room temperature at 4 cm^–1^ resolution. Power spectra were computed by Fourier transformation of 1000 (background; clean crystal surface) or 500 (sample) averaged interferograms. Absorbance spectra between 4000–800 cm^–1^ were then calculated from –log(sample intensities/background intensities). Cited frequencies are accurate to approximately 1 cm^–1^.

### Controlled rehydration of urea

3 µL of a 50 mM urea solution, prepared in double distilled water, were dried onto the crystal with a gentle stream of dry nitrogen gas at a flow rate of 300 mL/min. This led to loss of liquid water as evident from bands in the 3500–3100 and 1700–1600 cm^–1^ regions. Drying was continued until the absorbance spectrum was stable in the 1700–1400 cm^–1^ region. The sample was then allowed to equilibrate for several hours in atmospheres of controlled humidities, imposed by placing different saturated salt solutions (Table 1) in a well within a chamber that enclosed the sample (Figure S1). Spectra were recorded when the 1700–1400 cm^–1^ region had stabilized.^23^

**Table 1:** Salt solutions and relative humidities at room temperature.^23^

Saturated salt solution	Relative humidity at room temperature (%)
H_2_O (no salt)	100
KNO_3_	94
KCl	84
NH_4_SO_4_	81
NH_4_Cl	79
NaCl	75

### Serial Dehydration of Urea and Creatinine Solutions and Urine

3 µL of a 50 mM urea solution were dried onto the crystal and rehydrated by placing double distilled water in the humidification chamber described above. Once the spectrum had stabilized, the humidification chamber was removed and a series of spectra (each an average of 10 interferograms; approximately 4 s data acquisition time) were recorded whilst dry nitrogen gas was passed over the sample until the sample had stabilized (typically 50–100 spectra over 3–7 minutes). Rehydrated and dried spectra of 10 mM creatinine solutions were recorded in the same manner.

The same serial dehydration protocol was followed with two urine samples from healthy donors that were diluted × 4 with distilled water (to ensure that solute band intensities in the dried state did not exceed the instrument limit) before drying a 3 µL aliquot onto the crystal.

### Data Processing and Component Fitting

In general, the thickness of the samples was greater than two microns and, therefore, was greater than the depth of penetration (approximately 0.6 microns) and sufficient to fill the entire active volume above the prism surface. Hence, in order to adjust the ATR spectra for the distortion caused by the wavenumber-dependency of the pathlength,^24^ ATR data between 1510 and 1410 cm^–1^ were ramped before fitting of components by multiplying absorbance values by a factor of (wavenumber_max_/wavenumber) to allow for the inverse relation between effective pathlength and wavenumber. However, because the wavenumber range used for fitting is very narrow (100 cm^–1^), this correction is extremely small and, in practice, does not significantly alter the fitting parameters or the relative areas of the best fits.

Urea spectra were then decomposed using curve fitting analysis with combinations of Gaussian or pseudo-Voigt functions^25–28^ using Origin version 8.6 (OriginLab Corporation). A single full width at half-maximum (FWHM) was used to describe the combined Gaussian and Lorentzian components of the pseudo-Voigt function and *m*_u_ values were assigned to each component to define Lorentzian contributions. A linear baseline was also included, with offset and slope parameters allowed to vary during the fitting. With this protocol, four components were sufficient to fit all spectra at different stages of drying (see below). A similar protocol was used to decompose spectra between 1520 and 1470 cm^–1^ of rehydrated creatinine during drying using combinations of Lorentzian^25,26^ and pseudo-Voigt functions.

Combinations of these same four urea components, together with an additional component from creatinine at 1492 cm^–1^, were fitted to the 1510–1410 cm^–1^ region of spectra of urine at different stages of dehydration, again with baseline offset and slope parameters being allowed to vary during the fitting and with all other parameters fixed according to Tables 2 and 4.

**Table 2:** Parameters of components required for band analysis of creatinine spectra between 1520 and 1470 cm^–1^ corresponding to the (ν(C = N) (30) ν(CN) (27) *δ*(NCH) (24)) band.

Component centre (cm^–1^)	Function type	FWHM (cm^–1^)
1492	Lorentzian	24
1499	Lorentzian	29
1501	Gaussian	23

**Table 3:** Predicted frequencies of ν_as_(CN) band of urea in combination with water or other urea molecules. Calculations were performed using Gaussian 09.^29^ Where larger urea ensembles were modeled, only the frequencies of those ureas with the highest number of H-bonds to their carbonyl oxygen are listed.

Structure	Number of H-bonds to the carbonyl oxygen	Wavenumber (cm^–1^)
1 urea + 5 H_2_O	2	1466
1 urea + 2 H_2_O	2	1455
1 urea + 1 H_2_O	1	1420
1 urea	0	1386
2 ureas	1	1408, 1421
3 ureas	2	1432
4 ureas	3	1441
5 ureas	3	1445

**Figure 1:**
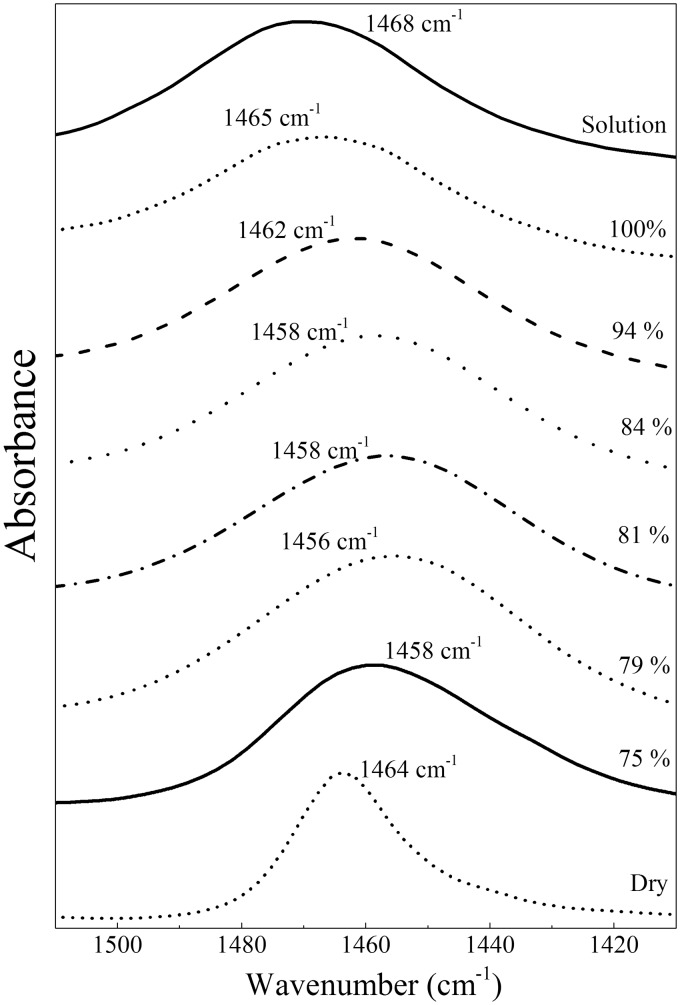
Spectra of urea between 1510 and 1410 cm^–1^ at fixed relative humidities. The ν_as_(CN) band peak at 1468 cm^–1^ in solution downshifts as relative humidity decreases to 79%, before upshifting towards the 1464 cm^–1^ position characteristic of dry urea at lower relative humidities (spectra are not shown to scale).

**Figure 2:**
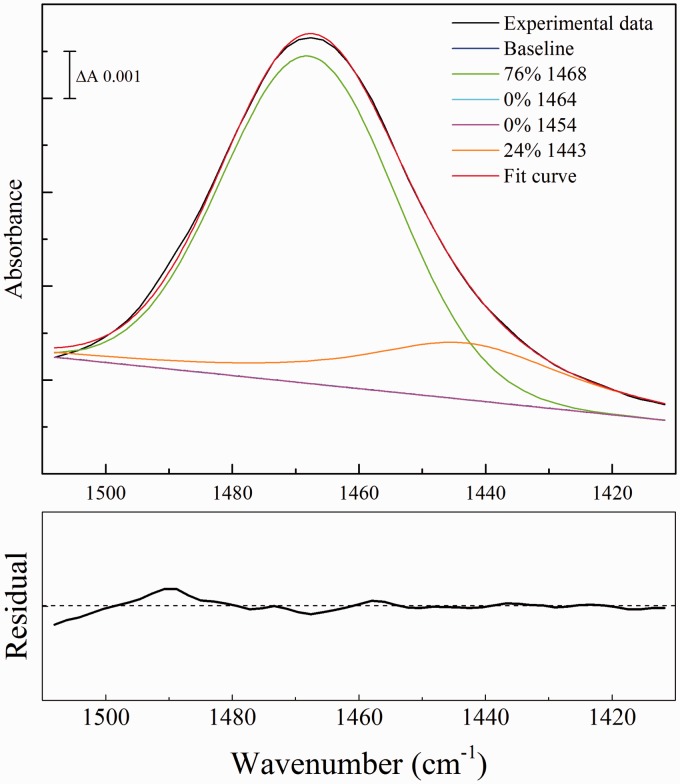
Component fitting to the ν_as_(CN) band of urea in solution. A spectrum of 200 mM urea in aqueous solution was recorded. Absorbance contributions of bulk water were first subtracted using an ATR spectrum of pure water and the 1510–1410 cm^–1^ region was then corrected for the effects of ramping caused by recording in ATR mode. An optimal fit was then made of intensities of the four components of fixed frequencies and bandwidths (Table 4), together with an unrestricted linear sloping baseline. Fractions of each component were calculated from the peak areas (integrated intensities). The residual for the overall fit is shown underneath at the same scale as the component fit, with the center line representing zero.

In cases of compounds in dilute solutions where bulk liquid water would have made a significant contribution to the spectral region used (solution spectrum of Figure 1 and Figure 2), the absorbance contributions of bulk water were removed by fractional subtraction of a spectrum of pure liquid water; optimal subtraction was assessed by minimization of the OH stretching modes in the 3400 cm^–1^ region and the combination band between 2200 and 2000 cm^–1^.

### Structure and Frequency Calculations

Infrared spectra of urea–water and urea–urea molecular ensembles were modeled. Firstly, nominal molecular models were built by stepwise addition of water or urea using the computer program Facio, version 18.6.2.^29^ Structures were then energy minimized using Gaussian 09 software^30^ with the B3LYP density functional in conjunction with the 6–31 G(d) basis set, using the UCL Legion supercomputer facility. Calculations were performed with a continuum water matrix using the SCRF = Water option (default dielectric constant = 78.3553). Vibrational spectra were then simulated with the energy-minimized structures. Simulated frequencies were visualized with Facio 18.6.2 after scaling^31^ with the Facio default factor of 0.9614. The predicted IR spectrum of creatinine was modeled in the same manner.

## Results and Discussion

### Kinetic Resolution of Multiple Urea Hydration States

**Figure 3:**
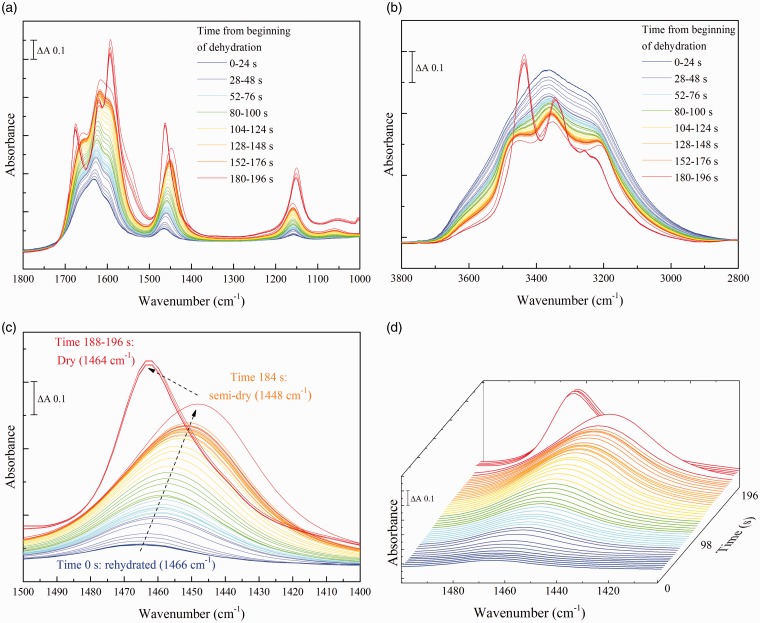
Serial spectra of a rehydrated urea sample during drying. 3 µL of a 50 mM solution of urea were firstly dried on the crystal and rehydrated in a water-saturated atmosphere. Spectra were then recorded during drying over an approx. three minute period. (a) the 1800–1000 cm^–1^ ‘fingerprint’ region; (b) the 3800–2800 cm^–1^ region. (c) and (d) two-dimensional and three-dimensional plots of the 1500–1400 cm^–1^ region showing the ν_as_(CN) band behavior. Rehydrated urea spectra are shown in blue and dried urea spectra in red.

As expected with a small molecule that forms multiple hydrogen bonds with water, the majority of bands of urea change their frequencies and relative intensities between the solution and fully dried states. Dehydration resulted in the loss of the broad absorbance bands of liquid water in the 1700–1600 cm^–1^ and 3700–2900 cm^–1^ ranges, with concurrent intensification of narrower bands of urea in these regions (Figures 3a and 3b). The rehydrated spectrum of urea is very similar to that of urea in solution and the fully dried spectrum is essentially the same as that of pure, crystalline urea (Figure S2a)^22,32^. However, the behavior was clearly not a simple transition between these two states. For example, the ν_as_(CN) band in the rehydrated spectrum was centered at 1466 cm^–1^, close to its position at 1468 cm^–1^ in the spectra of urea in solution at physiological concentrations (Figure S2b). As the sample became dehydrated this peak gradually downshifted, transiently reaching a minimum at 1448 cm^–1^ before rapidly upshifting to the 1464 cm^–1^ position that is characteristic of the fully dried state (Figures 3c and 3d).

### Stepwise Rehydration of Urea

By controlling the relative humidity of the surrounding atmosphere, it was possible to stabilize the intermediate partially hydrated forms of urea. Figure 1 summarizes the peak positions of the ν_as_(CN) band after stabilization at different relative humidities. As the relative humidity decreased, the peak (1468 cm^–1^ in solution) downshifted, settling at 1456 cm^–1^ at 79% humidity. However, as the relative humidity was further decreased, the peak upshifted towards the fully dried value of 1464 cm^–1^.

### Component Fitting to Urea Spectra

The ν_as_(CN) normal mode of urea is the only contributor to the 1510–1410 cm^–1^ region. Combinations of Gaussian and pseudo-Voigt components were fitted to this region of typical spectra obtained during the transition from hydrated to fully dried forms. Before fitting, a correction was made for the ramping of spectral intensities caused by recording in ATR mode (see Methods). Four components (Table 4) were required to adequately fit all spectra in this wavenumber range based on the root mean square error compared to a three or fewer component fit. Fitting with additional components did not improve the error of the fit.

**Table 4:** Parameters of components required for band analysis of urea spectra between 1510 and 1410 cm^–1^ corresponding to the ν_as_(CN) band.

Peak position of the urea ν_as_ (CN) band (cm^–1^)	Function type	*m*_u_ (fraction of Lorentzian component)	FWHM (cm^–1^)
1468	Gaussian	n/a	32
1464	Pseudo-Voigt	0.61	19
1454	Pseudo-Voigt	0.66	36
1443	Pseudo-Voigt	0.97	41

**Figure 4:**
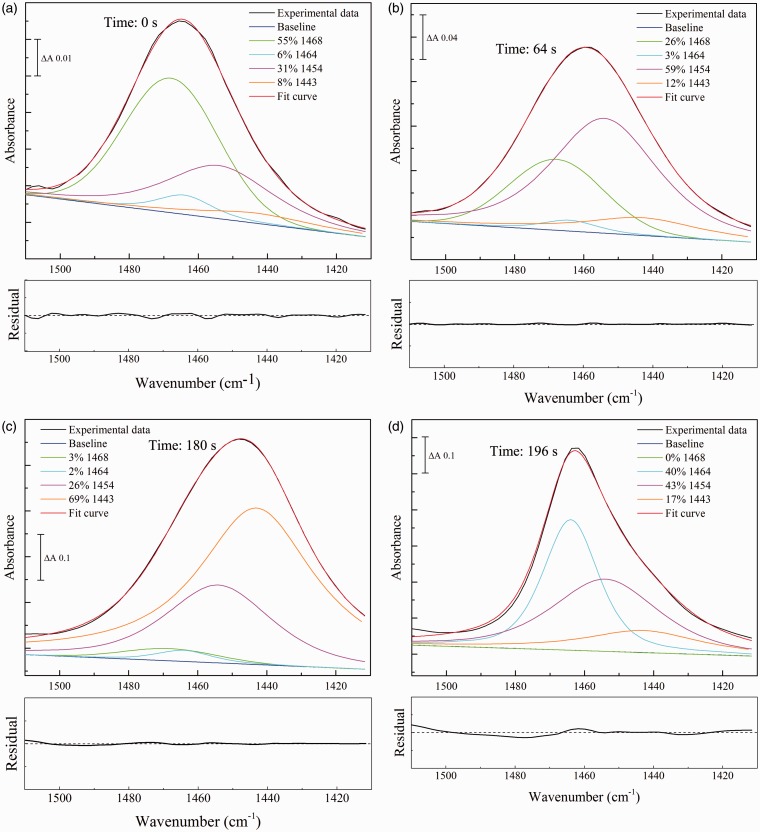
Component fitting to the ν_as_(CN) band of urea during the transition from rehydrated to dry states. Four spectra were selected from the series obtained during drying of rehydrated urea of Figure 1. Times from beginning of the dehydration are indicated on each panel. Before fitting, spectra were corrected for effects of ramping caused by recording in ATR recording mode, followed by optimal fitting of the intensities of the four components of fixed frequencies and bandwidths (Table 4), together with unrestricted linear sloping baselines. (a) rehydrated; (b) and (c) intermediate partially-hydrated; (d) dry states. The residuals for each overall fit are shown underneath each panel at the same scale as the corresponding component fit, with the center line representing zero (that in Figure 3a consists largely of a residual water vapor component).

The fitting data with these components are shown in Figure 4. For reference, the best fit of combinations of these same components to this region of urea in solution is shown in Figure 2; this is dominated by the Gaussian component centered at 1468 cm^–1^ that also dominates the rehydrated spectrum (Figure 4a). As the rehydrated sample dried, the 1468 cm^–1^ component diminished and components at 1454 and 1443 cm^–1^ emerged (Figure 4b). The 1443 cm^–1^ component became more dominant as the drying progressed (Figure 4c) before the band rapidly upshifted to the 1464 cm^–1^ form that is characteristic of fully dried urea (Figure 4d).

### Hydration States of Creatinine

**Figure 5:**
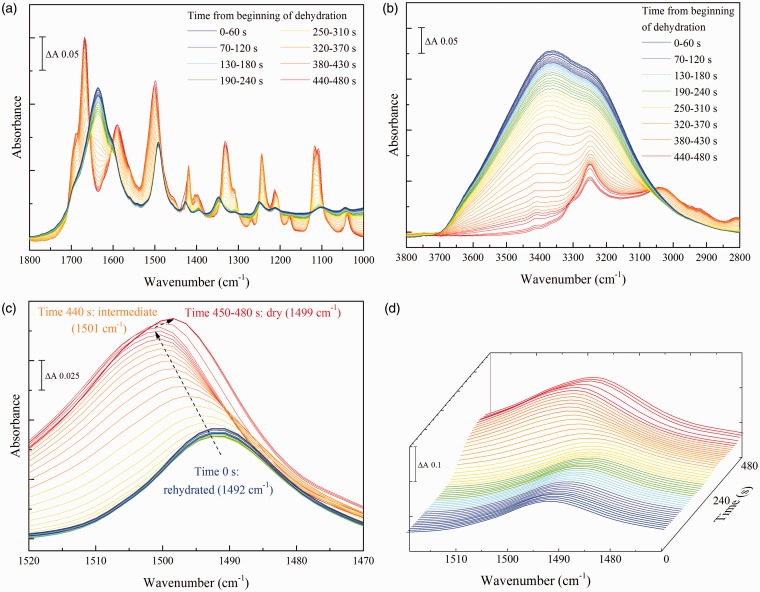
Serial spectra of a rehydrated creatinine sample during drying. 3 µL of a 10 mM solution of creatinine was firstly dried onto the crystal surface and rehydrated in a water-saturated atmosphere. Spectra were then recorded during drying over a three-minute period. (a) the 1800–1000 cm^–1^ ‘fingerprint’ region; (b) the 3800–2800 cm^–1^ region. (c) and (d) two-dimensional and three-dimensional plots of the 1520–1470 cm^–1^ region showing the (ν(C = N) (30) ν(CN) (27) δ(NCH) (24)) band behavior. Rehydrated creatinine spectra are shown in blue and dried creatinine spectra in red.

**Figure 6:**
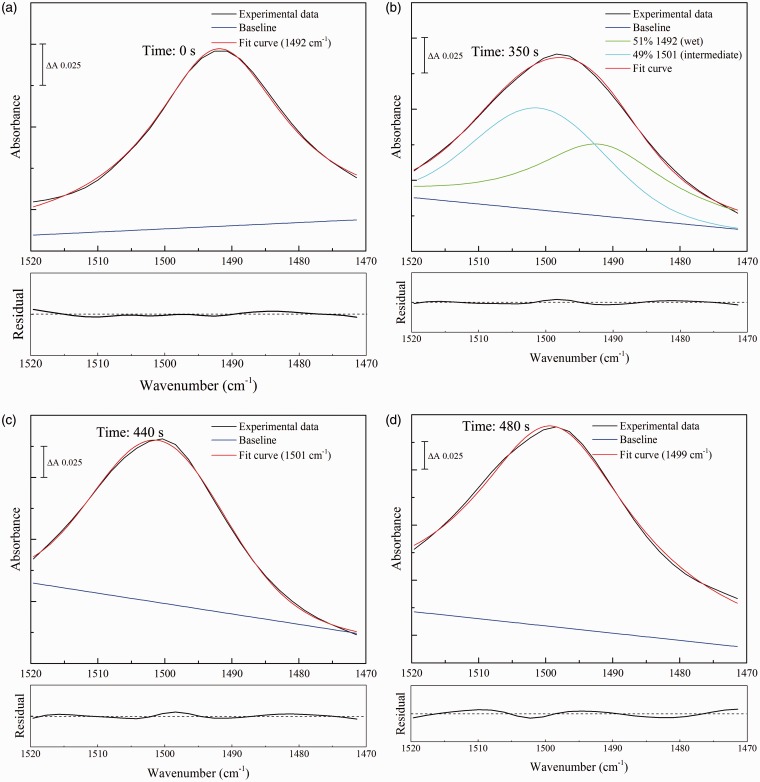
Curve fitting to the 1520 and 1470 cm^–1^ region of the FT-IR spectrum of creatinine during the transition from rehydrated to dry states. 3 µL of a 10 mM solution of creatinine were firstly dried onto the crystal surface and then rehydrated in a water-saturated atmosphere. Four spectra were selected from the series obtained during drying of this rehydrated sample. Spectra shown were corrected for effects of ramping caused by recording in ATR recording mode, followed by optimal fitting of the intensities of the three components of fixed wavenumbers and bandwidths (Table 2), together with unrestricted linear sloping baselines. (a) rehydrated; (b) and (c) intermediate partially-hydrated; (d) dry states. The residuals for each overall fit are shown underneath each panel at the same scale as the corresponding component fit, with the center line representing zero.

Hydration-dependent variations in absorption band frequencies and intensities occur with other urinary components, though none that were studied were as remarkable as those seen with urea. Creatinine is another major urinary component and has an absorption band at 1492 cm^–1^ in solution that partly overlaps with the ν_as_(CN) absorption band of urea. Based on previously published work^33,34^ and frequency predictions performed in Gaussian 09 of pure creatinine (see Methods), this band can be assigned to a single complex normal mode (ν(C = N) (30) ν(CN) (27) *δ*(NCH) (24)). Spectra of a rehydrated sample during drying were recorded (Figure 5) and components were fitted to the data between 1520 and 1470 cm^–1^ after correction for the ramping effect of recording in ATR mode. The 1492 cm^–1^ band upshifted during dehydration to an intermediate position at 1501 cm^–1^ (Figure 5c). Continued drying resulted in a slight downshift to 1499 cm^–1^, the same position as in dry, crystalline creatinine (not shown). In both solution and rehydrated states, this band can be approximately described by a single Lorentzian component centered at 1492 cm^–1^ with a FWHM of 24 cm^–1^ (Figure 6a and Table 2). After drying, or in crystalline creatinine, the band approximates to a single Lorentzian component at 1499 cm^–1^ with a FWHM of 29 cm^–1^ (Figure 6d). However, an additional Gaussian component at 1501 cm^–1^ with a FWHM of 23 cm^–1^ is required in order to adequately describe spectra in the partially hydrated conditions (Figure 6b). All spectra could be decomposed adequately with combinations of these three components.

### Hydrated Forms of Urea in Dried Urine Samples

**Figure 7:**
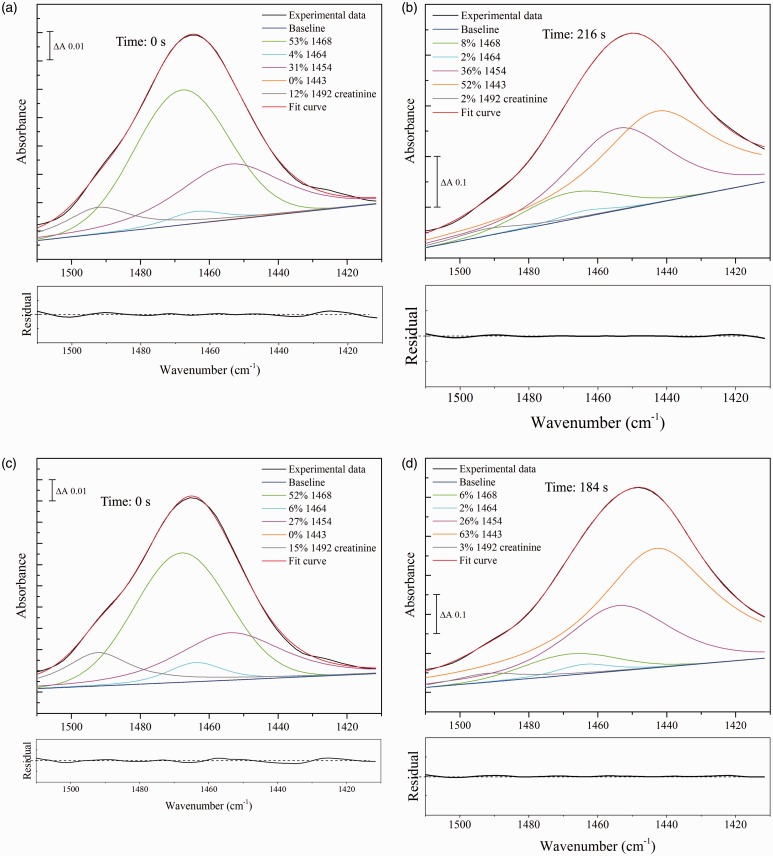
Fitting of urea and creatinine components to the 1510–1410 cm^–1^ region of FT-IR spectra of human urine. 3 µL samples of two typical healthy human urine samples were dried onto the crystal surface and rehydrated with a water-saturated atmosphere. Spectra were recorded after stabilization (a) rehydrated sample N1 and (c) rehydrated sample N2. Samples were then dried and spectra were rerecorded after they had stabilized (b) dried sample N1; (d) dried sample N2. The fractional contributions of each component were determined from peak areas (integrated intensities). The residuals for each overall fit are shown underneath each panel at the same scale as the corresponding component fit, with the center line representing zero.

The 1510–1410 cm^–1^ region of spectra of rehydrated human urine samples from healthy donors during drying was recorded. Two representative samples are shown in Figure 7. This region is dominated by the ν_as_(CN) band of urea, together with a smaller contribution from the 1492 cm^–1^ band of creatinine. Spectra were analyzed by fitting the four components of pure urea (Table 4) together with additional smaller contributions from the possible forms of creatinine (Table 2) and unrestricted linear sloping baselines.

In the rehydrated state, as expected, the urine spectrum is dominated by the 1468 cm^–1^ form of urea together with a 10–15% contribution from the 1492 cm^–1^ form of creatinine (Figure 7a and 7c). This is similar to that observed with fresh, liquid urine (Figure S3). However, even after drying by extensive exposure to the dry nitrogen flow, the urea components (Figure 7b and 7d) corresponded to those of its partially hydrated (1454 and 1443 cm^–1^) states (Figure 4c), rather than the 1464 cm^–1^ band of the fully dried form. Because the enhancement of the urea band intensity on drying is much greater than that of creatinine (compare Figures 3c and 5), the creatinine contributions to this region of the dried spectra become much smaller ( ≤ 3%). In Figure 7b and 7d, a single component at 1492 cm^–1^, corresponding to hydrated creatinine, appears adequate, consistent with water retention in the dried urine samples, though accurate assignment to its different states cannot be made with confidence because of its very low relative intensity.

**Figure 8:**
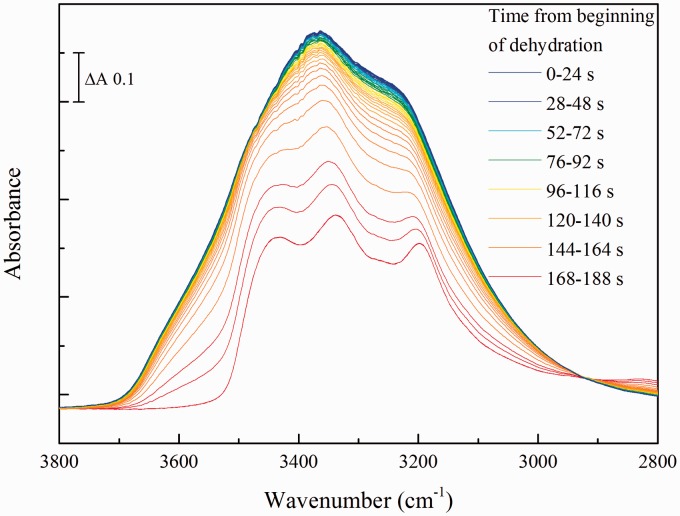
Behavior of the 3800–2800 cm^–1^ region of spectra of human urine during drying. The figure shows spectra of human urine sample, N2, when changing from the rehydrated (blue) into the dried (red) states.

The retention of intermediate hydration states of urea in dried urine samples is also clearly seen in the 3700–2900 cm^–1^ region of spectra of dried urine (Figure 8). Whereas it is clear that the broad band of liquid water has been lost, indicating that no major bulk liquid water remained, the remaining urea bands resembled those of the partially hydrated forms, rather than those of the fully dried states (Figure 3b). Hence, again, it is clear that drying had trapped predominantly the intermediate states of urea, rather than the fully dried form.

### Factors in Urine Affecting Observed Behavior

**Figure 9:**
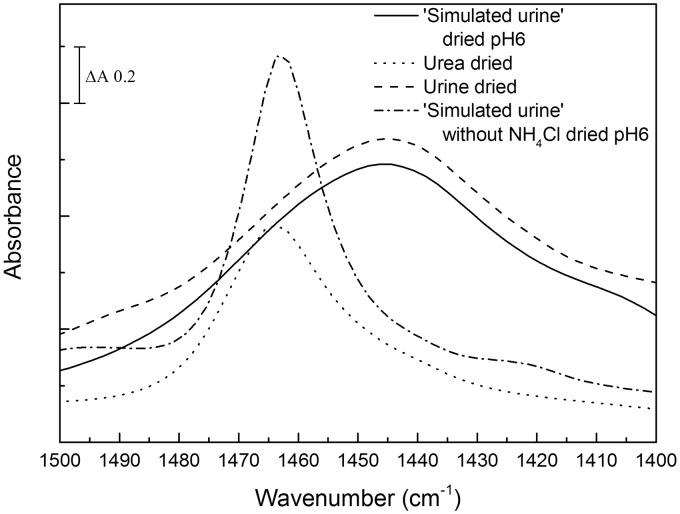
Behavior of ν_as_(CN) urea band in dried simulated urine. 3 µL of simulated urine containing 115 mM urea, 50 mM NaCl, 29 mM NH_4_Cl, 19 mM KH_2_PO_4_, 10 mM K_2_SO_4_ and 5 mM creatinine were dried onto the crystal surface and the spectrum was recorded after stabilization (solid line). Also shown are spectra of 3 µL 50 mM urea after drying (dotted line) and 3 µL healthy donor urine after drying (dashed).

The question arises as to what factor(s) control the different behaviors of urea when dried from pure solution versus urine. A “simulated urine” solution was prepared containing 115 mM urea, 50 mM NaCl, 29 mM NH_4_Cl, 19 mM KH_2_PO_4_, 10 mM K_2_SO_4_, and 5 mM creatinine, buffered to pH 6 with potassium hydroxide.^35^ When a 3 µL sample was dried onto the ATR crystal, the ν_as_(CN) band of urea stabilized around 1445 cm^–1^ (Figure 9), reproducing the behavior of urea in urine. However, the relative amount of the 1464 cm^–1^ component formed on drying increased when one of the major components (ammonium chloride, creatinine, or phosphate) was omitted, or if the mixture was diluted substantially before drying a 3 µL sample. In contrast, if one of these components was omitted but a replacement solute (e.g., sodium sulfate) was added to maintain the solute mass, then the urea again failed to form the 1464 cm^–1^ component. Furthermore, if urine samples were extensively diluted, then a significant fraction of the 1464 cm^–1^ form of urea appeared on drying. It is therefore concluded that the urinary effect is a non-specific one, caused simply by the mass of dried components (in urine, urea accounts for only 25–50 % of total solutes) creating a surface barrier that prevents complete dehydration of the material close to the crystal surface.

### Electronic Structure Modeling and Physical Basis of Intermediates

Changes in the spectra of pure urea during dehydration must arise from changes in urea–water and/or urea–urea interactions. A similar phenomenon has already been reported by Grdadolnik and Maréchal^13^ and Jung et al^18^ who observed a shift in the urea ν_as_(CN) band in very concentrated solutions; these were assigned to specific urea–urea and/or urea/water interactions in the limited water environments. In aqueous solution, urea interacts directly with four water molecules.^12–14^ Gaussian 09^30^ software was used to calculate the IR spectra of urea alone, in different hydration states and in interactions with other urea molecules. The predicted wavenumber of the ν_as_(CN) band (Table 3) downshifted from 1466 to 1386 cm^–1^ as the number of urea-bound water molecules decreased from 5 to 0. This downshift was due to the loss of H-bonding interactions, in particular between water hydroxyls and the carbonyl oxygen of urea.^13^ In support of this, the most dramatic predicted changes corresponded to the removal of the last two waters which, in our simulations, were those interacting with the carbonyl group. Conversely, the predicted wavenumber of the ν_as_(CN) band of anhydrous urea upshifted from 1386 to 1445 cm^–1^ as its carbonyl oxygen made an increasing number of H-bonds with adjacent urea –NH_2_ group(s) (Table 3). Indeed, in an ensemble of urea molecules, multiple ν_as_(CN) frequencies were predicted. However, the highest wavenumber correlated with those ureas with the greatest number of H-bonding interactions of their carbonyl oxygen. Although the precise peak positions predicted during the simulation vary from those observed in the experimental data, these calculations, at least qualitatively, support a proposal that the experimentally observed initial wavenumber downshift of the ν_as_(CN) band on drying is due to dehydration of urea, and the subsequent upshift arises from direct urea–urea interactions as the last waters are removed.

## Conclusions

In the context of IR analyses of urinary samples, at least four forms of urea with distinct IR spectra should be considered, with their relative amounts dependent on hydration level. These correspond to the rehydrated form (equivalent to the solution form), a dried form and two additional partially hydrated forms. Additional hydration states of creatinine can also be formed, though the IR differences between them are much weaker than those of urea.

Spectra of dried urine samples show that the partially hydrated states of urea tend to become trapped in the dense matrix of dried urinary components, with a predominance of the lowest wavenumber (1443 cm^–1^) form, preventing direct urea–urea interactions. The retention of such partially hydrated forms with altered IR signatures complicates the analysis of IR spectra of dried samples such as urine, and it is important that they are recognized in diagnostic analyses of dried biological tissues and fluids. However, the limited number of distinct forms can easily be accommodated in decomposition procedures for quantitative analyses, as shown here with the contributions of different states of urea in dried urine samples.

## Supplementary Material

Supplementary material
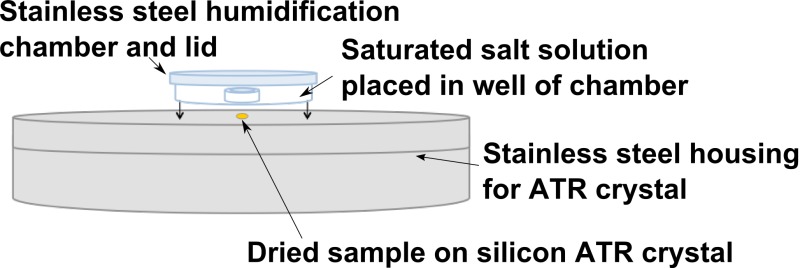

Supplementary material
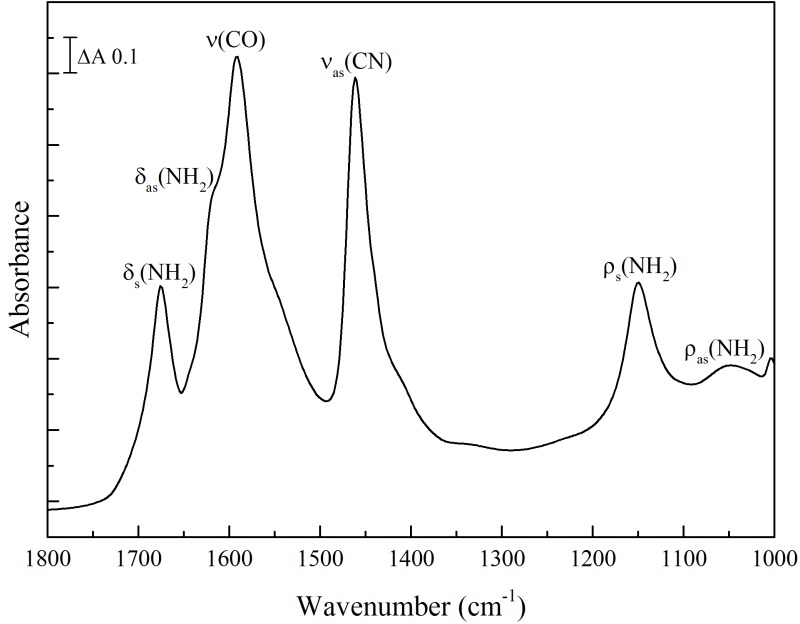

Supplementary material
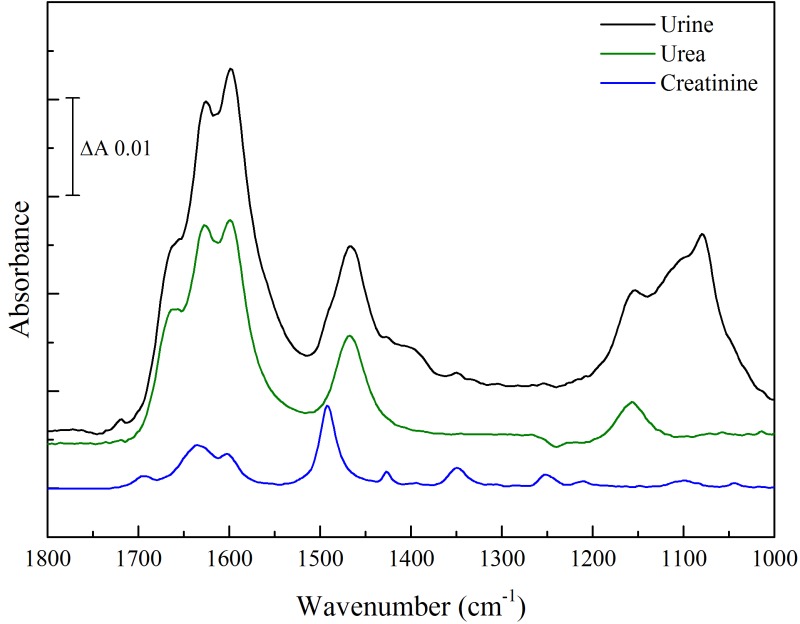

## Supplemental Material

All supplemental material mentioned in the text, including three figures, is available in the online version of the journal at http://asp.sagepub.com/supplemental

## References

[1] Diem M. Modern Vibrational Spectroscopy & Micro-Spectroscopy, Chichester, UK: John Wiley & Sons, 2015.

[2] Moss D. Biomedical Applications of Synchrotron Infrared Microspectroscopy, Cambridge, UK: Royal Society of Chemistry, 2011.

[3] Petibois C. Gionnet K. Gonçalves M. Perromat A. Moenner M. Déléris G. “Analytical Performances of FT-IR Spectrometry and Imaging for Concentration Measurements within Biological Fluids, Cells, and Tissues”. Analyst 2006; 131: 640–647. 16633577 10.1039/b518076g

[4] Petibois C. Rigalleau V. Melin A.-M. Perromat A. Cazorla G. Gin H. Determination of Glucose in Dried Serum Samples by Fourier-Transform Infrared Spectroscopy. Clin. Chem 1999; 45(9): 1530–1535. 10471657

[5] Budínová G. Salva J. Volka K. Application of Molecular Spectroscopy in the Mid-Infrared Region to the Determination of Glucose and Cholesterol in Whole Blood and in Blood Serum. Appl. Spectrosc 1997; 51(5): 631–635.

[6] Hoşafçi G. Klein O. Oremek G. Mäntele W. Clinical Chemistry Without Reagents? An Infrared Spectroscopic Technique for Determination of Clinically Relevant Constituents of Body Fluids. Anal. Bioanal. Chem 2007; 387: 1815–1822. 17089104 10.1007/s00216-006-0841-3

[7] Shaw R.A. Low-Ying S. Leroux M. Mantsch H.H. Toward Reagent-Free Clinical Analysis: Quantitation of Urine Urea, Creatinine, and Total Protein from the Mid-Infrared Spectra of Dried Urine Films. Clin. Chem 2000; 46(9): 1493–1495. 10973898

[8] Petrich W. Dolenko B. Früh J. Ganz M. Greger H. Jacob S. Disease Pattern Recognition in Infrared Spectra of Human Sera with Diabetes Mellitus as an Example. Appl. Opt 2000; 39(19): 3372–3379. 18349906 10.1364/ao.39.003372

[9] Heise H. Voigt G. Lampen P. Küpper L. Rudloff S. Werner G. Multivariate Calibration for the Determination of Analytes in Urine Using Mid-Infrared Attenuated Total Reflection Spectroscopy. Appl. Spectrosc 2001; 55: 434–443.

[10] Pérez-Guaita D. Sánchez-Illana Á. Garrigues S. de la Guardia M. Determination of Lidocaine in Urine at Low ppm Levels using Dispersive Microextraction and Attenuated Total Reflectance–Fourier Transform Infrared Measurements of Dry Films. Microchem. J 2015; 121: 178–183.

[11] Stumpe M.C. Grubmüller H. Aqueous Urea Solutions: Structure, Energetics and Urea Aggregation. J. Phys. Chem. B 2007; 111: 6220–6228. 17497766 10.1021/jp066474n

[12] Lee C. Stahlberg E.A. Fitzgerald G. Chemical Structure of Urea in Water. J. Phys. Chem 1995; 99: 17737–17741.

[13] Grdadolnik J. Maréchal Y. Urea and Urea–Water Solutions—an Infrared Study. J. Mol. Struct 2002; 615: 177–189.

[14] Nandel F.S. Verma R. Singh B. Jain D.V.S. Mechanism of Hydration of Urea and Guanidium Ion: A Model Study of Denaturation of Proteins. Pure Appl. Chem 1998; 70(3): 659–664.

[15] Rezus Y.L.A. Bakker H.J. Effect of Urea on the Structural Dynamics of Water. Proc. Natl. Acad. Sci. U. S. A 2006; 103(49): 18417–18420. 17116864 10.1073/pnas.0606538103 PMC1693679

[16] Carr J. Buchanan L.E. Schmidt J.R. Zanni M.T. Skinner J.L. Structure and Dynamics of Urea/Water Mixtures Investigated by Vibrational Spectroscopy and Molecular Dynamics Simulation. J. Phys. Chem. B 2013; 117(42): 13291–13300. 23841646 10.1021/jp4037217 PMC3808478

[17] Finer E.G. Franks F. Tait M.J. Nuclear Magnetic Resonance Studies of Aqueous Urea Solutions. J. Am. Chem. Soc 1972; 94(13): 4424–4429.

[18] Jung Y.M. Czarnik-Matusewicz B. Bin Kim S. Characterization of Concentration-Dependent Infrared Spectral Variations of Urea Aqueous Solutions by Principal Component Analysis and Two-Dimensional Correlation Spectroscopy. J. Phys. Chem. B 2004, pp. 13008–13014.

[19] Tanaka H. Nakanishi K. Touhara H. Computer Experiments on Aqueous Solutions. VII. Potential Energy Function for Urea Dimer and Molecular Dynamics Calculation of 8 mol % Aqueous Solution of Urea. J. Chem. Phys 1985; 82(11): 5184–5191.

[20] Hoccart X. Turrell G. Raman Spectroscopic Investigation of the Dynamics of Urea–Water Complexes. J. Chem. Phys 1993; 99(11): 8498–8503.

[21] Idrissi A. Molecular Structure and Dynamics of Liquids: Aqueous Urea Solutions. Spectrochim. Acta A 2005; 61: 1–17. 10.1016/j.saa.2004.02.039 15556415

[22] Keuleers R. Desseyn O. Rousseau B. Van Alsenoy C. Vibrational Analysis of Urea. J. Phys. Chem. A 1999; 103: 4621–4630.

[23] Greenspan L. Humidity Fixed Points of Binary Saturated Aqueous Solutions. J. Res. Natl. Bur. Stand., Sect. A 1977; 81A(1): 89–96.

[24] Goormaghtigh E. Raussens V. Ruysschaert J.-M. Attenuated Total Reflection Infrared Spectroscopy of Proteins and Lipids in Biological Membranes. Biochim. Biophys. Acta 1999; 1422: 105–185. 10393271 10.1016/s0304-4157(99)00004-0

[25] Bradley M.S. Krech J.H. High-pressure Raman Spectra of the Acetone Carbonyl Stretch in Acetone-Methanol Mixtures. J. Phys. Chem 1993; 97: 575–580.

[26] Knapp E.W. Fischer S.F. The Concentration Dependence of the Vibrational Linewidth and Shift in Liquid Binary Mixtures: An Analytical Model. J. Chem. Phys 1982; 76: 4730–4735.

[27] Stancik A.L. Brauns E.B. A Simple Asymmetric Lineshape for Fitting Infrared Absorption Spectra. Vib. Spectrosc 2008; 47: 66–69.

[28] Sánchez-Bajo F. Cumbrera F.L. The Use of the Pseudo-Voigt Function in the Variance Method of X-Ray Line-Broadening Analysis. J. Appl. Crystallogr 1997; 30(4): 427–430.

[29] Suenaga M. Facio: New Computational Chemistry Environment for PC GAMESS. J. Comput. Chem., Jpn 2005; 4(1): 25–32.

[30] Frisch M.J. Gaussian 09, revision A.1, Wallingford, USA: Gaussian Inc., 2009.

[31] Tantirungrotechai Y. Phanasant K. Roddecha S. Surawatanawong P. Sutthikhum V. Limtrakul J. Scaling Factors for Vibrational Frequencies and Zero-Point Vibrational Energies of Some Recently Developed Exchange-Correlation Functionals. J. Mol. Struct 2006; 760(1-3): 189–192.

[32] Rousseau B. Van Alsenoy C. Keuleers R. Desseyn H.O. Solids Modeled by Ab-Initio Crystal Field Methods. Part 17. Study of the Structure and Vibrational Spectrum of Urea in the Gas Phase and in its P42_1_*m* Crystal Phase. J. Phys. Chem. A 1998; 102(32): 6540–6548.

[33] Trendafilova N. Kurbakova A.P. Efimenko I.A. Mitewa M. Bontchev P.R. Infrared Spectra of Pt(II) Creatinine Complexes. Normal Coordinate Analysis of Creatinine and Pt(creat)_2_(NO_2_)_2_. Spectrochim. Acta 1991; 47 A(5): 577–584.

[34] Bayrak C. Bayari S.H. Vibrational and DFT Studies of Creatinine and its Metal Complexes. Hacet. J. Biol. Chem 2010; 32(2): 107–118.

[35] Rose C. Parker A. Jefferson B. Cartmell E. The Characterization of Feces and Urine: a Review of the Literature to Inform Advanced Treatment Technology. Crit. Rev. Environ. Sci. Technol 2015; 45(17): 1827–1879. 26246784 10.1080/10643389.2014.1000761 PMC4500995

